# Triploid Hybrid Vigor in Above-Ground Growth and Methane Fermentation Efficiency of Energy Willow

**DOI:** 10.3389/fpls.2022.770284

**Published:** 2022-02-23

**Authors:** Dénes Dudits, András Cseri, Katalin Török, László Sass, Zoltán Zombori, Györgyi Ferenc, Péter Poór, Péter Borbély, Zalán Czékus, Radomira Vankova, Petre Dobrev, Judit Szántó, Zoltán Bagi, Kornél L. Kovács

**Affiliations:** ^1^Institute of Plant Biology, Biological Research Centre, Eötvös Loránd Research Network (ELKH), Szeged, Hungary; ^2^Department of Plant Biology, University of Szeged, Szeged, Hungary; ^3^Department of Biological Resources, Centre for Agricultural Research, Agricultural Institute, Martonvásár, Hungary; ^4^Institute of Experimental Botany, Czech Academy of Sciences, Prague, Czechia; ^5^UBM Feed Zrt. Pilisvörösvár, Hungary; ^6^Department of Biotechnology, University of Szeged, Szeged, Hungary

**Keywords:** growth rate, plant hormones, CO_2_ fixation, water use, biogas, *Salix*

## Abstract

Hybrid vigor and polyploidy are genetic events widely utilized to increase the productivity of crops. Given that bioenergy usage needs to be expanded, we investigated triploid hybrid vigor in terms of the biology of biomass-related willow traits and their relevance to the control of biomethane production. To produce triploid hybrid genotypes, we crossed two female diploid Swedish cultivars (Inger, Tordis) with two male autotetraploid willow (*Salix viminalis)* variants (PP-E7, PP-E15). Field studies at two locations and in two successive years recorded considerable midparent heterosis (MPH%) in early shoot length that ranged between 11.14 and 68.85% and in the growth rate between 34.12 and 97.18%. The three triploid hybrids (THs) developed larger leaves than their parental cultivars, and the MPH% for their CO_2_ assimilation rate varied between 0.84 and 25.30%. The impact of hybrid vigor on the concentrations of plant hormones in these TH genotypes reflected essentially different hormonal statuses that depended preferentially on maternal parents. Hybrid vigor was evinced by an elevated concentration of jasmonic acid in shoot meristems of all the three THs (MPH:29.73; 67.08; 91.91%). Heterosis in auxin-type hormones, such as indole-3-acetic acid (MPH:207.49%), phenylacetic acid (MPH:223.51%), and salicylic acid (MPH:27.72%) and benzoic acid (MPH:85.75%), was detectable in the shoots of TH21/2 plants. These hormones also accumulated in their maternal Inger plants. Heterosis in cytokinin-type hormones characterized the shoots of TH3/12 and TH17/17 genotypes having Tordis as their maternal parent. Unexpectedly, we detected abscisic acid as a positive factor in the growth of TH17/17 plants with negative MPH percentages in stomatal conductance and a lower CO_2_ assimilation rate. During anaerobic digestion, wood raw materials from the triploid willow hybrids that provided positive MPH% in biomethane yield (6.38 and 27.87%) showed negative MPH in their acid detergent lignin contents (from –8.01 to –14.36%). Altogether, these insights into controlling factors of above-ground growth parameters of willow genotypes support the utilization of triploid hybrid vigor in willow breeding to expand the cultivation of short rotation energy trees for renewable energy production.

## Introduction

Climate change interacts with the plant kingdom in a complex way. First, plants, as sessile organisms, are the primary victims of extreme and damaging environmental conditions. Simultaneously, they play a positive role in climate change mitigation and renewable energy production. Forest management practices provide different ways to improve protection against climatic impacts, namely *via* afforestation, reforestation, reduced deforestation, and increasing carbon density as an indicator for forest carbon sink capacities (Canadell and Raupach, 2008). Different forest ecosystems and management practices should be evaluated for their forestation potential to insure high C sequestration capacity into both biomass and soil stocks. In this respect, shrub willows grown as woody crops have outstanding potential to serve as an optimal feedstock to produce bioenergy, biofuels, and bioproducts with environmental and rural development benefits (Clifton-Brown et al., 2019). Besides, traditional forestry, short rotation forestry (SRF) that entails a high density of plants and frequent harvesting can offer an alternative silvicultural system to be harnessed for climate change mitigation and phytoremediation goals (refer to the review by Capuana, 2020).

Willow species (*Salix* spp.) are fast-growing trees that offer several advantages for establishing short rotation plantations. The primary issue in such systems is the biomass yield which depends on several factors, such as the crop’s genotype, soil condition, water availability, management practice, and rotation cycle. Willow plants can be grown on marginal cropping lands, and they can play a significant role in maintaining a positive greenhouse gas (GHG) balance due to their proven potential to fix and accumulate carbon during their vegetative growing phase (Hammar et al., 2017). As reviewed by Djomo et al. (2011), short-rotation woody crops such as poplar (*Populus* spp.) and willow yield 14.1–85.9 times more energy than does coal (ER_*coal*_∼0.9) per unit of fossil energy input, resulting in GHG emissions that were 9–161 times lower than those of coal (GHG_*coal*_∼96.8). Yet, it is important to recognize that the increase in forest-provided bioenergy can generate both benefits and risks when the net exchange of carbon between the atmosphere and forested ecosystems is duly considered (Favero et al., 2020).

Willow plants cultivated for energy production (energy willow) can serve as a raw material for cellulosic ethanol production (reviewed by Dey et al., 2020). Earlier, Gaykawad et al. (2013) reported the production of 11.5 g/L of ethanol’s concentration from willow wood chips after concentration of ethanol by hydrophobic pervaporation. Further, the energy of a willow-based pyrolysis system for biomethane production can contribute to enhanced energy performance and a negative global warming potential (Ahmadi Moghaddam et al., 2019). Recently, Kakuk et al. (2021) showed that green willow biomass harvested from shrubs, younger than 1 year, could also function as a very efficient biogas substrate. Beyond climate change, pollution of soil and water by toxic heavy metals or by organic pollutants generates serious problems that require global actions, including the use of woody species for land decontamination (Capuana, 2020; Hauptvogl et al., 2020). Willow plantations are capable of heavy metal and organic compound phytoremediation that is accompanied by gains in biomass production (Wani et al., 2020).

For such multipurpose applications of short rotation, *Salix* plantations to succeed will require specific cultivars, namely those with augmented traits according to technology and product demands. Hence, willow breeding techniques are gaining significance and garnering more attention (reviewed by Hanley and Karp, 2014). The broad diversity of the genus *Salix*, harboring 330–500 species, with more than 200 hybrids now developed, offers useable starting material for targeted breeding programs (Isebrands and Richardson, 2014). Similar, to natural events, like the formation of new species (Suda and Argus, 1968), hybridization and polyploidization events are also essential methodologies for willow breeding. Crosses between the tetraploid *S. miyabeana*, native to Japan, Korea, and China, and the diploid *S. purpurea* or its hybrids resulted in triploid genotypes distinguished by higher green biomass than either their diploid or tetraploid parental species (Serapiglia et al., 2014). In those studies, the highest-yielding genotype was a triploid hybrid (*S. koriyanagi* × *S. purpurea* × *S. miyabeana*). These interspecies triploid hybrids (THs) displayed significant heterosis for harvestable biomass and biomass-related growth traits in both greenhouse and field settings (Carlson and Smart, 2021). The detailed characterization of autotetraploid willow plants demonstrated positive effects of chromosome set duplication on several key agronomic and environmental traits (Dudits et al., 2016).

Since improving biomass yield and energy production efficiency is now a central goal (Kulig et al., 2019), we have initiated a crossing program between two leading Swedish diploid cultivars (Tordis and Inger) and our autotetraploid genotypes. We postulated that a novel genetic composition based on triploidy and heterosis could result in a substantial improvement in the biology of willow plants used for bioenergy production. The aim of this paper was to monitor triploid heterosis with respect to a suite of traits, such as biomass, growth rate, photosynthetic CO_2_ uptake, evaporation, carbohydrate content, and hormonal status of shoots. As a unique possibility, we characterize the genotype-dependent levels of methane production to elucidate the roles and relevance of triploid hybrid vigor for biogas generation.

## Materials and Methods

### Plant Material

A set of autotetraploid [polyploid Energo (PP-E); 2*n* = 4*x* = 76] genotypes was generated by colchicine treatment of axillary buds from Energo plants (*Salix viminalis* var. Energo diploid cultivar). The detailed characterization of these genotypes in comparison to the Energo plants was published earlier (Dudits et al., 2016). Using tetraploid plants, a crossbreeding program was initiated to produce triploid genotypes. As greenhouse crossings, the receptive female catkins of commercial energy willow cultivars (i.e., Inger and Tordis) were hand-pollinated with pollen grains collected from male flowers of the autotetraploid plants (PP-E7 and PP-E15). After crossing, hybrid seeds were germinated as *in vitro* cultures, and their genome size was determined by flow cytometry of nuclei isolated from their root tips. Vegetative propagation of the selected triploid hybrid lines was carried out in a breeding garden by using cuttings of woody stems.

### Field Experiments for Quantification of Triploid Heterosis in Shoots’ Early Growth

In the spring of 2019, woody stems of the 2-year-old parental and triploid hybrid plants were cut back, and the daily growth rates of their newly developing shoots were determined over a 37-day-period from five plants by measuring three shoots per plant. Ten plants per genotype were grown in randomized plots of our breeding garden Szeged, Hungary [location I: global positioning system (GPS) coordinates: 46°15′10.8′′N, 20°8′53.66′′E]. Next spring in 2020, woody stems of the 1-year-old parental and triploid hybrid plants were cut back, and the daily growth rates of their newly developing shoots in 4 days were determined from 10 plants (measuring three shoots per plant). Twenty plants per genotype were grown in randomized plots of the breeding garden in Kiskunhalas, Hungary (location II. GPS coordinates: 46° 26′ 7.5876′′ N and 19° 29′ 0.3408′′ E). To characterize hybrid vigor, we calculated the mid-parent heterosis (MPH%) as the heterosis over mid-parent (MP%) = [(F1-MP)/MP × 100], where F1 is the numerical value trait measurement in the hybrid and MP values are the mean values of the parents (P1 + P2)/2. In addition, we present heterosis values relative to the cultivar parents (CPH%) = [(F1-CP)/CP × 100] for the selected traits.

Plants were annually harvested in winter during the dormant phase. We measured the woody biomass of 60 cm-long cuttings and collected woody stem samples for their chemical analysis of lignocellulose compositions and biogas fermentation potential.

### Phenotyping of Parental and Triploid Hybrid Willow Plants for Quantification of Leaf Size, Photosynthetic Parameters, and Water Use

Willow plants were grown in the soil of the complex stress diagnostic system (Cseri et al., 2020) under greenhouse conditions (at 21°C and illuminated with ∼400 μmol photons m^–2^ s^–1^). The analyzed plants were grown under an optimal water supply (60% soil water content) during the whole life cycle, and their water consumption data were stored automatically by the computer.

To measure the photosynthetic functioning, both stomatal conductance (g_*sw*_; mol H_2_O m^–2^ s^–1^) and CO_2_ assimilation rate (A_*N*_; μmol CO_2_ m^–2^ s^–1^) were monitored in young, fully expanded leaves with a portable photosynthesis system (LI-6400, LI-COR, Inc., Lincoln, NE, United States) and an atmospheric CO_2_ source, according to Poór et al. (2011). The CO_2_ concentration was maintained at 400 ppm by a soda-lime reagent (Sigma-Aldrich ACS reagent). On each leaf, 2 cm^2^ of its area was measured with the controlled CO_2_ flow (300 μmol mol^–1^) at 25°C under greenhouse conditions.

The carbohydrate (soluble sugars and starch) concentration was determined according to Poór et al. (2011). Briefly, 100 mg [fresh weight (FW)] of leaf material was ground in liquid nitrogen, from which soluble sugars were extracted with 1 ml of 80% ethanol at 80°C for 30 min. The homogenate was centrifuged at 2,600 g for 10 min. After a second extraction step, the combined supernatants were used to determine the soluble sugar content at 630 nm after its reaction with anthrone (Normapur, VWR Int., Leuven, Belgium) dissolved in 72% sulfuric acid, where glucose (Normapur, VWR Int., Leuven, Belgium) dissolved in 80% ethanol was then used as the standard. The remaining pellet was washed with 1 ml of deionized water, then hydrolyzed with 1 ml of 1.1% HCl at 100°C for 30 min and centrifuged for 10 min at 2,600 g. The starch concentration was also evaluated spectrophotometrically at 630 nm with anthrone reagent, and for this purpose starch (Normapur, VWR Int., Leuven, Belgium) dissolved in 1.1% HCl served as the standard.

### Quantification of Mid-Parent Heterosis% or Cultivar-Parent Heterosis (CPH%) in Hormonal Status of Triploid Willow Plants Grown *in vitro*

Woody stem cuttings (each 10 cm in length) of triploid hybrid and parental genotypes were placed into holes of a plastic foam floating in a pool filled with the Knop-solution. The dormant buds were activated, and the outgrowing shoots grew in a thermostat room at 21°C under artificial light. The level of illumination was ∼200 μmol photons m^–2^s^–1^. To determine hormone contents (pmol g^–1^ FW) in the shoot tip meristems, tissues samples (30–80 mg fresh weight) were purified and analyzed by following the methodology of Dobrev and Kamínek (2002) and Dobrev and Vankova (2012) as described in our previous publication (Dudits et al., 2016). The shoot tip tissues were collected from two replicates of plants whose mean value is provided.

### Analysis of Primary Cell Wall Components (Cellulose, Hemicellulose, and Lignin) in Wood Samples

For the determination of fiber composition on a dry weight basis, the wood samples were dried at 55°C in an air dryer for 12–16 h. After drying, each sample was ground to pass through a 1-mm forage mill. Analysis of fibers was then carried out following the protocols described by Van Soest and Robertson (1985). The acid detergent fiber (ADF) was determined gravimetrically, as the amount of residue remaining upon ignition after receiving the 72% H_2_SO_4_ treatment. Hemicellulose% was estimated as neutral detergent fiber (%)—ADF (%). Cellulose (%) was estimated as ADF (%)—lignin (%). Three biological duplicates of wood samples per genotype were used in each fiber analysis.

### Batch Anaerobic Digestions for Methane Production

The batch experiments were carried out in 160-ml glass reactors in triplicates. The milled wood particles were first inoculated with a fresh sample from an industrial scale mesophilic biogas plant, then fed with pig slurry and maize silage mix (Zöldforrás Biogas Plant, Szeged, Hungary), and finally filtered through a 2-mm mesh. The CH_4_ content was measured by an Agilent 6,890 N gas chromatograph (Agilent Technologies, Santa Clara, United States). Triplicate batch digesters were assembled for each combination of substrates having a substrate–inoculum ratio (S/I) of 0.5, for which the reaction temperature was set at 37°C. Full details of the fermentation protocol are provided in our recent article (Kakuk et al., 2021).

### Statistical Analyses

Prior to statistical analyses, statistically significant outliers (if there were any) were identified by the extreme studentized deviate (ESO) method using the graph pad online outlier calculator. The significant outliers were removed from the dataset if the normal distribution was confirmed without the outliers detected by the ESO test. Statistical analyses were implemented by SigmaPlot 12.0 software (Systat Software Inc.), the significant differences among genotypes were evaluated by using Duncan’s multiple range test after one-way ANOVA if all the preconditions for the test were met. If any of the preconditions were not met, then the Student–Newman–Keuls test (SNK test) or Dunn’s multiple comparisons test was used after ANOVA on ranks as indicated in the figure captions. Differences were considered significant if *p* ≤ 0.05.

## Results

### Crossing the Autotetraploid With the Diploid Energy Willow Plants Generated in Triploid Hybrids

In the greenhouse, the receptive female catkins of the Inger and Tordis cultivars were hand-pollinated with pollens collected from male flowers of Energo tetraploid plants (PP-E 7 and PP-E15). Embryos were excised and further cultured *in vitro*, and outgrowing plantlets were propagated in agar cultures. The hybrid and parental plants were first planted into the soil of pots and grown in the greenhouse. Cuttings of woody stems were rooted in water and used for nuclei isolation from their root tips and characterized by flow cytometry. Based on these DNA content indicators, we were able to establish a collection of triploid hybrid (TH) lines, all of which were propagated by the cutting of woody stems in an outside nursery during the study years.

### Triploid Heterosis Expressed in Shoot Growth Rate and Stem Parameters Under Field Conditions

In the third year of the willow plantation, woody stems were cut back in spring. Their newly developed shoots were used to characterize the genotype-dependent early growth and stem parameters of willow plants, and this was done between May 5 and June 11, 2020 (Table 1, location I). Depending on the crossing combinations, significant differences in shoot length and growth rate could be recorded between the parental plants and THs. Both Swedish cultivars (Inger, Tordis) and the two tetraploid lines (PP-E7 and PP-E15) showed differences in shoot length and daily growth rate in June 2020. Crossing the fast-growing Inger with the tetraploid PP-E7 plants led to reduced growth in THs (TH21/2), exhibiting increased growth intensity. In this crossing combination, in June 2020, we detected an MPH value of 37.88% for shoot length and one of 42.96% for growth rate. A similar hybridization effect was recorded in the TH17/17 hybrids with heterosis for shoot length (43.46% MPH) and growth rate (39.57% MPH) values in the June measurements. Combining the two parental genotypes that have low growth rates (cultivar Tordis and PP-E7) generated considerable hybrid vigor in shoot length (54.73% MPH) and growth rate (60.55% MPH) in the TH3/12 plants sampled in June 2020. Comparing the MPH percentages determined at the two measurement time points, the heterosis of shoot length evidently was decreased in the TH17/17 genotype, while MPH% values increased in plants of the TH3/12 and the TH21/2 lines. This pattern suggested the possibility of increasing the expression of heterosis in the later period vegetative growth for these two genotypes. The diameter values in the growing phase of the green stems differed significantly between the two cultivars, and the autotetraploid genomes (PP-E7 and PP-E15) had developed wider stems (Table 1). For this trait, triploid heterosis was expressed in all three hybrid genotypes as follows: TH3/12: MPH: 38.98%; TH17/17: MPH: 42.34%, and TH21/2: MPH:29.71%.

**TABLE 1:** Expression of varying triploid heterosis for key shoot growth parameters of willow in crossing combinations of diploid Swedish cultivars with autotetraploid genotypes under two field sites.

Geno-types	Year 2020 Location I.				Year 2021 Location II.		
	Shoot length (cm)		Growth rate/day (mm)	Stem diameter (mm) in June	Shoot length (cm)		Growth rate/day (mm)
	May 5th	June 11th			April 27th	May 1st	
**Tordis**	27.50 ± 6.05 d	95.33 ± 17.27 c	18.27 ± 3.73 b	4.94 ± 0.94 c	50.67 ± 10.30 Bc	82.60 ± 14.14 b	7.98 ± 2.01 b
**TH3/12** **MPH%**	46.53 ± 3.22 b **42.81**	158.80 ± 16.67 a **54.73**	30, 34 ± 4.12 a **60.55**	9.13 ± 1.37 b **38.98**	71.70 ± 9.46 a **49.53**	141.59 ± 20.03 a **68.85**	17.48 ± 4.04 a **97.18**
**PP-E 7**	37.67 ± 1.51 c	109.93 ± 17.17 c	19.53 ± 4.35 b	8.20 ± 0.30 b	45.23 ± 5.10 C	85.11 ± 9.14 b	9.75 ± 1.56 b
**Tordis**	27.50 ± 6.05 d	95.33 ± 17.27 c	18.27 ± 3.73 b	4.94 ± 0.94 c	50.67 ± 10.30 Bc	82.60 ± 14.14 b	7.98 ± 2.01 b
**TH17/17** **MPH%**	48.93 ± 1.99 ab **53.64**	146.03 ± 7.86 ab **43.76**	26, 24 ± 2.51 ab **39.57**	9.19 ± 0.63 b **42.34**	56.11 ± 5.35 b **11.14**	118.31 ± 11.86 a **37.15**	15.27 ± 1.95 a **70.71**
**PP-E 15**	36.20 ± 3.72 c	107.83 ± 24.51 bc	19, 33 ± 5.94 b	7.98 ± 0.96 b	50.30 ± 5.50 Bc	89.93 ± 11.85 b	9.91 ± 2.18 b
**Inger**	49.93 ± 3.10 ab	154.50 ± 15.49 a	28, 23 ± 4.77 a	8.87 ± 0.97 b	67.31 ± 6.55 A	130.56 ± 13.47 a	15.66 ± 2.48 a
**TH21/2** **MPH%**	52.00 ± 5.65 a **18.72**	178.33 ± 9.34 a **37.88**	33, 59 ± 0.70 a **40.66**	11.07 ± 0.55 a **29.71**	75.63 ± 8.75 a **34.41**	146.63 ± 25.02 a **35.98**	17.04 ± 3.84 a **34.1**2
**PP-E 7**	37.67 ± 1.51 c	109.93 ± 17.17 c	19, 53 ± 4.35 b	8.20 ± 0.30 b	45.23 ± 5.10 C	85.11 ± 9.14 b	9.75 ± 1.56 b

*The midparent heterosis (MPH%) was calculated as heterosis over the midparent (MP%) = [(F1-MP)/MP × 100], where F1 is the numerical value trait measurement in the hybrid and MP values are the mean values of the parents (P1 + P2)/2. Results are mean ± SD. Values denoted with different letters show significant differences at P ≤ 0.05 (Duncan’s or Dunn’s test). Bold values highlight the MPH% and CPH% information.*

In 2021, the same growth characteristics were recorded in late April by examining shoots developed in the second year of the willow plantation (Table 1, location II). Considerable hybrid vigor in plant height and growth rate was evident in all three crossing combinations under these distinct conditions. Crossing the Tordis plants with PP-E7 autotetraploid resulted in the highest MPH for shoot length. This was found in TH3/12 plants (MPH:68.85%), and likewise for daily growth rate (MPH: 97.18%). In general, these second-year data collected under better field conditions indicate that triploid hybrid vigor in terms of growth rate was higher than in the preceding year, except plants in TH21/2.

**FIGURE 1:**
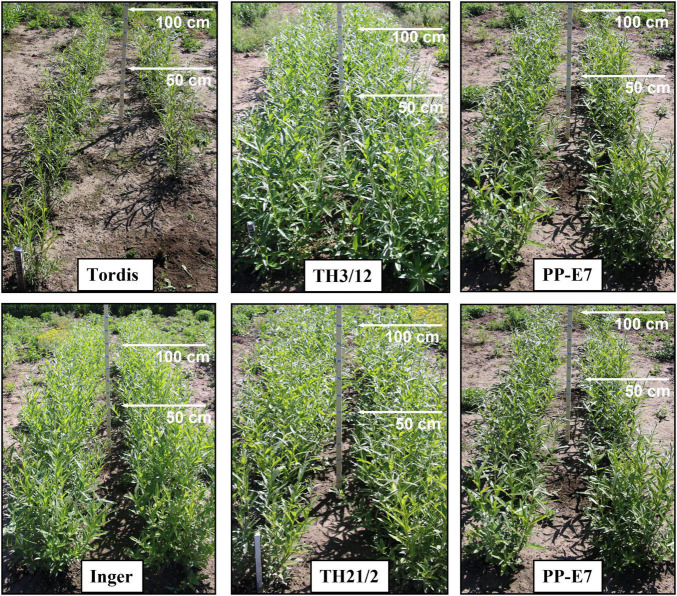
Growth habit and green biomass characteristics show hybrid vigor in triploid hybrids (TH3/12, TH21/2) vis-à-vis two diploid Swedish cultivars (Tordis, Inger) and two autotetraploid parents (PP-E7, PP-E15). Pictures were taken in spring 2021 of plants grown at location II.

Early growth vigor of triploid hybrid willow plants could be recorded under field conditions. As shown in Figure 1, the TH3/12 plants produced substantially more biomass than did the parental plants (Tordis, PP-E7). Although the TH21/2 triploid hybrid plants accrued a slightly greater amount of the green biomass than did the leading Swedish cultivar (Inger), they were nonetheless considerably more productive than their tetraploid parental plants (PP-E7).

### Triploid Hybrid Willow Genotypes Show Increased Leave Biomass, Net Photosynthetic CO_2_ Uptake, Evaporation, Carbohydrate Content in Comparison With Leading Cultivars

**FIGURE 2:**
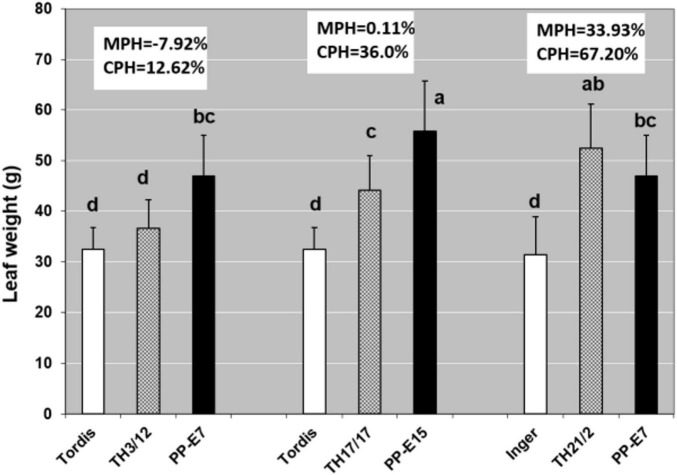
The triploid hybrid energy willow (TH) lines had statistically significant heterosis for leaf weight in relation to the cultivar parents (CPH%) under greenhouse conditions. Statistically, significant increases relative to the TH3/12 and TH17/17 hybrids were detected in the case of tetraploid parents (PP-E7 and PP-E15). Heterosis over the cultivar parent was calculated as (CPH%) = [(F1-CP)/CP × 100]. Results are Means + SD, *n* = 10. Values denoted with different letters show significant differences at *p* ≤ 0.05 (Duncan’s test).

On searching for the physiological basis of detected hybrid vigor, leaf functions are considered suitable primary targets. Differences in leaf sizes can also be expressed by weight data (Figure 2). Doubling the genome size resulted in the largest leaves, which could have contributed to the lack of MPH%, except in the TH21/2 hybrid. When the THs were compared with the commercial cultivars (Inger, Tordis), the CPH% indicated significant levels of hybrid vigor for all THs but especially for the TH21/2 plants.

**FIGURE 3:**
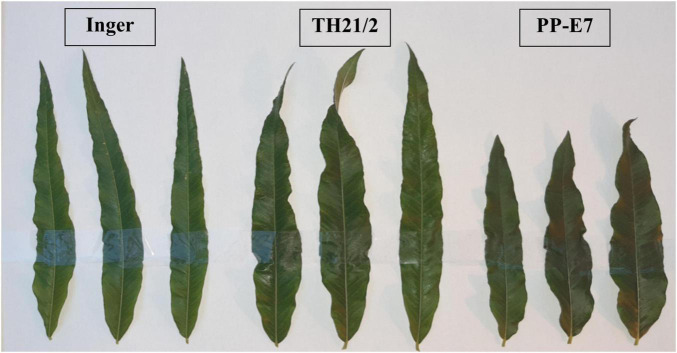
Leaves from triploid hybrids (TH21/2) combine the morphological traits of parental leaves (Inger, PP-E7). Plants were grown in the greenhouse.

The length and width of leaves differed significantly between the parental and TH21/2 plants (Figure 3). The two parents produced leaves with different morphologies, with shortened but wider leaves characteristic of tetraploid plants (PP-E 7). By contrast, in the triploid hybrid TH21/2 plants this is clearly a feature of the combination of long and wide leaf characteristics.

It was shown previously that a positive correlation between photosynthetic rate and biomass production could only be detected in a restricted set of willow genotypes (Andralojc et al., 2014). Still, willow breeding programs could consider photosynthetic capacity as a promising target to increase biomass production. Hence, we tested the expression of hybrid vigor in traits related to that (Table 2).

**TABLE 2:** Expression of midparent heterosis (MPH%) and cultivar-parent heterosis (CPH%) for leaf functions is genotype-dependent.

Genotypes	CO_2_ assimilation rate (μmol CO_2_ m^–2^ s^–1)^	Stomatal conductance (mol H_2_O m^–2^ s^–1)^	Water use efficiency (g green biomass/L)	Sucrose mg/gFW	Starch mg/gFW
**Tordis**	4.69 ± 1.90c	0.0357 ± 0.0254ab	28.67 ± 0.81ns	9.78 ± 0.97c	8.83 ± 0.85bc
**TH3/12** **MPH%** **CPH%**	8.27 ± 2.08a **25.30** **76.33**	0.0683 ± 0.0239ab **72.04** **91.32**	29.32 ± 1.81ns **3.55** **2.27**	9.42 ± 0.59c **–7.42** **–3.68**	12.19 ± 0.58a **19.86** **38.05**
**PPE-7**	8.51 ± 2.20a	0.0437 ± 0.0254ab	27.96 ± 1.57ns	10.57 ± 0.75c	11.51 ± 0.73a
**Tordis**	4.69 ± 1.90a	0.0357 ± 0.0254ab	28.67 ± 0.81ns	9.78 ± 0.97c	8.83 ± 0.85a
**TH17/17** **MPH%** **CPH%**	6.58 ± 1.48b **0.84** **40.30**	0.0377 ± 0.0168ab **–33.63** **5.60**	27.79ns **–4.21** **–3.07**	9.96 ± 0.72b **0.96** **1.84**	10.70 ± 0.33abc **10.48** **21.18**
**PPE-15**	8.36 ± 1.45a	0.0779 ± 0.0336a	30.51 ± 4.01ns	9.95 ± 0.39c	10.54 ± 0.75ab
**Inger**	8.44 ± 1.97a	0.0321 ± 0.0232b	26.68 ± 2.94ns	7.91 ± 0.79d	6.92 ± 0.53c
**TH21/2** **MPH%** **CPH%**	9.26 ± 1.08a **9.26** **9.72**	0.0543 ± 0.0190ab **43.27** **69.16**	26.83 ± 2.59ns **–0.56** **–1.79**	11.58 ± 1.77a **25.32** **46.40**	11.77 ± 1.57a **27.73** **70.09**
**PPE-7**	8.51 ± 2.20a	0.0437 ± 0.0254ab	27.96 ± 1.57ns	10.57 ± 0.75c	11.51 ± 0.73a

*Parental and hybrid willow genotypes were grown in soil in the greenhouse. The MPH% was calculated as heterosis over mid-parent (MP%) = [(F1-MP)/MP × 100], where F1 is the numerical value trait measurement in the hybrid and MP values are the mean values of the parents (P1 + P2)/2. In this table presents heterosis relative to the cultivar parents: (CPH%) = [(F1-CP)/CP × 100]. Results are Means ± SD. Values denoted with different letters show significant differences at P ≤ 0.05 (Duncan’s; Dunn’s test or SNK test). Bold values highlight the MPH% and CPH% information.*

In agreement with our study, it was found that tetraploid plants were capable of higher photosynthetic efficiency (Dudits et al., 2016), and in the present greenhouse study, both PP-E7 and PP-E15 plants were capable of high net photosynthetic CO_2_ uptake. Based on this a low MPH was recorded for the TH3/12 and TH21/2 hybrid plants. The stomatal conductance values indicated a higher evaporation rate than in their parental plants. Considerable hybrid vigor could be seen in these crossing combinations. Table 2 also provides pertinent information on water requirements, as inferred from stomatal conductance values of willow plants with different genetic backgrounds. The two diploid cultivars exhibited relatively reduced levels of transpiration. Significant differences between the THs raise the possibility for selecting drought-tolerant energy willow genotypes. The highest positive MPH value for stomatal conductance was measured in plants of the TH3/12 hybrid. The TH21/2 hybrids expressed positive heterosis in this trait, yet low heterosis was found in water-use efficiency (MPH:0.56%; CPH:-1.79%). As presented in Table 2, the autotetraploid energy willow parental plants (PP-E15) exhibited the highest stomatal conductance and water use efficiency values. These parameters have contributed to the negative heterosis of stomatal conductance in the TH17/17 hybrid plants.

The primary products of photosynthesis are soluble carbohydrates which form starch, which is an insoluble and non-structural carbohydrate. The present set of energy willow genotypes let us analyze hybrid vigor in terms of the production of these compounds (Table 2). Sucrose concentration showed positive heterosis in the leaves of TH21/2 hybrids (MPH:25.31%; CPH:46.40%). Limited heterosis could be detected for starch accumulation, namely in leaves from the TH3/12 and TH21/2 hybrid plants.

### Hormonal Background of Triploid Heterosis in Early Shoot Growth *in vitro*

In short-rotation energy plants, the growth rate and later biomass formation are the key parameters that are governed by the hormonal status of the shoots. In this study, stem cuttings from the THs and their parents were cultured in the Knop-solution, and the outgrowing shoots were used to quantify a large set of plant growth regulators. In Tables 3A–C the concentrations of hormones that indicated genotype-dependent, variable MPH (MPH%) in shoots of different triploid hybrid plants when compared to their parents.

**TABLE 3:** Variation of the midparent heterosis (MPH%) in early shoots of willow plants grown in the Knop-solution from the triploid hybrid genotypes compared to their parental plants.

(A)			
*In vitro* culture in Knop-solution	**TORDIS**	**TH3/12**	**PP-E7**
Shoot height (cm)	4.30 bc	5.70 b	10.60 a

**Hormones (pmol g^–1^ fresh weight)**			

**Jasmonic acid** MPH%	173.08	298.17 **91.91**	137.66
**Jasmonate isoleucine** MPH%	4.29	7.38 **102.19**	3.01
**Abscisic acid** MPH%	623.75	817.14 **2.62**	968.82
**IAA-aspartate** MPH%	81.27	130.72 **3.92**	170.30
**Isopentenyl adenosine** MPH%	5.64	6.96 **43.95**	4.03
**Dihydrozeatin riboside** MPH%	2.41	7.09 **158.29**	3.08
***Cis*-zeatin riboside** MPH%	7.53	12.44 **72.42**	6.9
**Total cytokinin** **ribosides** MPH%	41.07	46.14 **24.38**	33.12
***Trans*-zeatin riboside -O-glucoside** MPH%	2.35	11.19 **86.97**	9.62

**(B)**			

*In vitro* culture in Knop-solution	**TORDIS**	**TH17/17**	**PP-E15**
Shoot height (cm)	4.30 bc	10.30 a	10.70 a

**Hormones (pmol g^–1^ fresh weight)**			

**Jasmonic acid** MPH%	173.08	264.23 **67.08**	143.21
**Abscisic acid** MPH%	623.75	812.07 **41.59**	523.30
**Dihydrophaseic acid** MPH%	100.99	120.12 **80.05**	32.44
**ABA-glucose ester** MPH%	64.36	71.39 **51.14**	30.11
**Neophaseic acid** MPH%	11.73	18.28 **61.13**	10.96
**IAA-glutamate** MPH%	0.00	2.70	0.00
**Isopentenyl adenosine** MPH%	5.64	6.16 **20.90**	4.55
***Trans*-zeatin riboside monophosphate** MPH%	1.12	8.82 **500.00**	1.82
**Isopentenyl adenosine monophosphate** MPH%	2.15	3.89	0.00

**(C)**			

*In vitro* culture in Knop-solution	**INGER**	**TH21/2**	**PP-E7**
Shoot height (cm)	2.70 c	5.90 b	10.60 a

**Hormones (pmol g^–1^ fresh weight)**			

**Indole-3-acetic acid** MPH%	242.29	732.87 **207.49**	234.39
**Phenylacetic acid** MPH%	90.10	199.70 **223.51**	33.36
**Salicylic acid** MPH%	1939.04	1730.20 **27.72**	770.38
**Benzoic acid** MPH%	2263.83	2971.87 **85.75**	935.95
**Jasmonic acid** MPH%	180.58	206.43 **29.73**	137.66
**Phaseic acid** MPH%	200.01	232.25 **15.24**	203.07
**ABA-glucose ester** MPH%	47.25	88.31 **42.11**	77.03
***Trans*-zeatin riboside** MPH%	29.07	26.17 **8.66**	19.10
**Dihydrozeatin riboside** MPH%	8.15	6.41 **14.16**	3.08
***Cis*-zeatin riboside** MPH%	6.84	8.72 **26.93**	6.9
***Trans*-zeatin riboside -O-glucoside** MPH%	14.48	12.42 **3.07**	9.62
***Cis*-zeatin-O-glucoside** MPH%	13.33	18.66 **35.36**	14.24

*The MPH% was calculated as heterosis over mid-parent (MP%) = [(F1-MP)/MP × 100], where F1 is the numerical value trait measurement in the hybrid and MP values are the mean values of the parents (P1 + P2)/2. Values denoted with different letters show significant differences at p ≤ 0.05 (Duncan’s test). Bold values highlight the MPH% and CPH% information.*

From the view of hybrid vigor, positive MPH could be detected for jasmonic acid (MPH: 91.91, 67.08, 29.73%)

in all three crossing combinations. Regarding the hormone composition in young shoots, the TH21/2 triploid hybrid plants differed significantly from the two other hybrids (TH17/17 and TH3/12) in having a high concentration of auxin-type hormones (Table 3C). In the TH21/2 shoots, levels of significant positive MPH were found in indole-3-acetic acid (MPH: 207.49%), phenylacetic acid (MPH:223.51%), salicylic acid (27.72%), and benzoic acid (85.75%). By contrast, expression of MPH for cytokinin-related compounds reached higher values in the shoots of TH3/12 and TH17/17 hybrid plants, especially in TH3/12 in comparison with the TH21/2 plants; for the TH3/12 shoots: isopentenyladenosine (43.95%), *cis*-zeatin riboside (72.42%), *trans*-zeatin riboside-*O*-glucoside (86.97%), and dihydrozeatin riboside (158.29%) and in TH17/17 shoots: isopentenyladenosine (20.90%) and *trans*-zeatin riboside monophosphate (500.00%, Tables 3A,B). Despite the complexity of young shoots’ hormonal status, the presented snapshot pointed to some key regulators involved in MPH.

### Can Heterosis Influence the Composition of Woody Biomass in Energy Willow?

The efficiency of anaerobic digestion (AD) of lignocellulose for biomethane production is directly determined by the compositional properties of wood biomass (recently reviewed by Xu et al., 2019). Accordingly, we also analyzed the possible involvement of hybrid vigor in determining wood tissue weight (g/cm) and the ratios of the most significant components of the woody cell wall (Table 4). Relatively low MPH was expressed for wood weight after crossing Inger and the PP-E7 parents (MPH:8.98%). Lignin is the most effective factor limiting the biodegradability of lignocellulose in the three-dimensional network inside the cell wall (Thomsen et al., 2016). Of the three THs, lower lignin contents were present in wood tissues from stems of the TH21/2 and TH3/12 genotypes, with negative MPH values (–14.36 and –8.01%, respectively). In stark contrast, the analyzed data revealed a positive MPH value (15.59%) for acid detergent lignin content from the wood of the TH17/17 hybrid. In the AD process, the amounts and polymerization degree of the cellulose component also play a central role. As evinced by Table 4, portions of cellulose in wood samples from the TH hybrids were intermediate between the cultivars and tetraploid parents. This indicated a significant increase in the hemicellulose content of wood samples from the TH3/12 THs. Conceivably, this could influence methane fermentation efficiency (refer below).

**TABLE 4:** Substrate characteristics of wood samples from parental and triploid willow genotypes and their positive and negative midparent heterosis (MPH%) values for cell wall components.

Genotypes	Wood weight (g/cm)	Acid detergent lignin(%)	Cellulose + cutin (%)	Hemicellulose (%)
Tordis	0.62 ± 0.01	15.18 ± 1.06bc	41.81 ± 2.13a	18.33 ± 0.39b
**TH3/12** MPH%	0.82 ± 0.07 **14.25**	13.89 ± 3.50c **–8.01**	37.93 ± 3.29ab **–1.48**	20.32 ± 0.42a **17.73**
PP-E7	0.80 ± 0.08	15.02 ± 1.09bc	35.19 ± 0.76b	16.19 ± 0.54d
Tordis	0.62 ± 0.01	15.18 ± 1.06bc	41.81 ± 2.13a	18.33 ± 0.39b
**TH17/17** MPH%	0.78 ± 0.05 **7.57**	18.09 ± 0.25ab **15.59**	35.60 ± 2.01a **–6.68**	18.10 ± 0.36b **–2.09**
PP-E15	0.83 ± 0.01	16.12 ± 3.03abc	34.49 ± 2.25b	17.13 ± 0.24c
Inger	0.88 ± 0.22	19.17 ± 0.24 a	39.53 ± 3.07 ab	17.19 ± 0.30c
**TH21/2** MPH%	0.92 ± 0.30 **8.98**	14.64 ± 1.64 bc **–14.36**	38.68 ± 3.81 ab **3.53**	16.19 ± 0.14d **–3.00**
PP-E7	0.80 ± 0.08	15.02 ± 1.09bc	35.19 ± 0.76b	16.19 ± 0.54d

*Results are mean ± SD. Values denoted with different letters show significant differences at p ≤ 0.05 (Duncan’s test). Bold values highlight the MPH% and CPH% information.*

### Crossing Combination-Dependent Triploid Heterosis in Biogas Fermentation Efficiency by Using Woody Stems of Energy Willow

**FIGURE 4:**
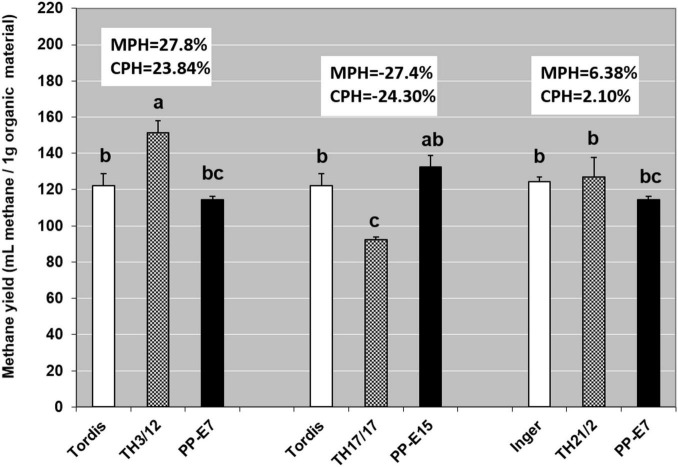
Genotype-dependent positive or negative triploid hybrid vigor in methane fermentation productivity from woody tissues. Values denoted with different letters show significant differences at *p* ≤ 0.05 (Duncan’s test).

**FIGURE 5:**
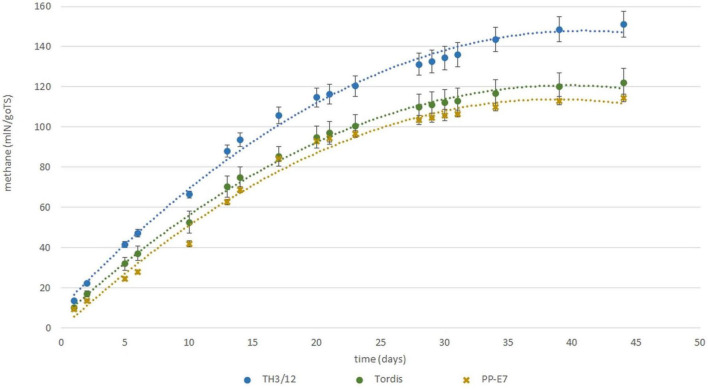
Expression of hybrid vigor for biomethane fermentation potential using woody samples from triploid hybrid (TH3/12) plants in comparison with parental plant samples during a complete cycle of the fermentation process.

Recent work by Kakuk et al. (2021) and Nyári et al. (2021) reported that the efficiency of using both woody and green biomass of willow as raw material for the anaerobic digestion (AD) and biomethane production depended on the genome size of willow plants. With that in mind, here, we evaluated whether the expression of triploid hybrid vigor in willow biomass-related traits could influence the biogas yield. As seen in Figure 4, methane yields when using woody tissues from the Swedish cultivars were the same, whereas using wood samples from the tetraploid genotypes led to moderate differences in the fermentation responses. For methane yield as a trait, the MPH varied from –27.40 to 27.80%, and a significantly positive value was detected in the fermentation of wood tissues from the TH3/12 THs. Biogas yield from the TH21/2 triploid wood also surpassed that of its parental plants but with lower heterosis. Hybrid vigor in terms of biomethane production from TH3/12 wood samples was detected during the whole fermentation cycle as shown by its cumulative values (Figure 5). The negative heterosis for methane yield from the wood samples of the TH17/17 hybrids originated from specific wood structural attributes, such as its higher lignin content (Table 4).

## Discussion

### The New Approach to Developing Triploid Hybrids of Energy Willow

Generation of hybrid vigor by crossing plants coupled with multiplying their genome size *via* polyploidy offers an efficient approach to optimize traits of forest trees, especially those that are grown in short-rotation systems. The wider use of *Salix* species for efficient bioenergy production depends on sufficient biomass productivity and promising techniques in fermentation. Biologically, there is a need to increase the genetic capacities for both biomass yield and the optimal composition of raw material. Together, these efforts could contribute to addressing global issues, such as climate change or environmental pollution. Previously, we reported on several traits of autotetraploid willow genotypes as their larger leaf and root systems, greater net photosynthetic CO_2_ uptake, improved photosynthetic functions, slower primary growth, and increased shoot diameter could jointly determine biomass yield (Dudits et al., 2016). In the present study, these tetraploid plants were crossed with two leading diploid Swedish cultivars to exploit positive effects arising from both hybrid vigor and polyploidy. This experimental approach demonstrates a novel way forward characterizing the interaction between hybrid vigor and polyploidy, which has been extensively studied for alloploid plants derived from hybridization between different species followed by chromosome doubling events (refer to review by Chen, 2010). Study by Carlson and Smart (2021) reported greater total above-ground dry biomass for a suite of interspecific *Salix* THs in comparison with either diploid or tetraploid parents.

### Triploid Hybrid Vigor in Terms of Growth Rates, Leaf Size, and CO_2_ Assimilation Traits

The present crossings with different parents resulted in a variable degree of MPH% in biomass-related traits in the THs. In all crossing combinations, examined here, considerable MPH values were recorded for early shoot length and daily growth rate under field or laboratory conditions (Tables 1, 3A–C). Despite the differing MPH% values between genotypes characterized in two growing seasons and at two locations, the ranking hybrid vigor of various crossings was similar across the different experimental conditions. In the Knop-solution as an optimal condition, tetraploid parents (PP-E7 and PP-E15) grew taller shoots than did triploid hybrid plants with reduced MPH% (Tables 3A–C). Our findings are in agreement with a study that found that environmental factors are capable of modulating the manifestation of hybrid vigor (Li et al., 2018). When evaluating the relationship between daily growth rate and actual plant height, stem diameter is a useful variable as noted for other woody species (Gleason et al., 2018). As Table 1 shows, plants of the Inger cultivar with the highest shoot growth rate attained maximum plant height and stem diameter. At the same time, the MPH% in its hybrid TH21/2 was relatively low. By contrast, the plants of the TH3/12 hybrids generated by crossing Tordis with PP-E7 willow were found to express significant hybrid vigor relative to the parental values.

Larger leaf size can be considered a significant contributing component to heterosis (Liu et al., 2020). In the present study, MPH for leaf size was detectable only in plants from the TH21/2 triploid hybrid (MPH:33.99%). However, when leaf weight values from all the three hybrids were compared with their cultivar parents, the CPH values provided clear evidence of heterosis (TH3/12:12.62%; TH17/17:36.00%; TH21/2:67.20%). This greater leaf area enabled a greater production of photosynthates. Further, the triploid willow hybrids featured a more efficient CO_2_ assimilation rate, especially relative to the Tordis parents (Table 2). Metabolome and proteome studies on maize hybrids and their inbred parents have shown positive MPH in the photosynthetic pathway (Li et al., 2020). Our study of willow clearly shows heterotic responses of different degrees in the CO_2_ assimilation rate, as well as in sucrose concentration and of starch accumulation in leaves of various triploid hybrid genotypes. In parallel, our data did not uncover direct relationships between these parameters. This finding might be due to a technical limitation caused by the different timing of CO_2_ assimilation rate measurements vs. the collection of leaf samples. In rice, heterotic responses were found to be related to the circadian rhythm pathway (Shen et al., 2015).

### Parental Combination-Dependent Negative or Positive Mid-Parent Heterosis in Stomatal Conductance

Water shortage and drought are the major concerns for plants generated by ongoing climate changes. Therefore, the water-use parameters of crop plants, including those trees used in agroforestry, can determine their end-use capacity for bioenergy production. Stomatal conductance values indicated positive MPH for the TH3/12 (72.04%) and TH21/2 (43.27%) hybrids. These higher evaporation rates could increase the drought sensitivity of these THs that might be compensated in part by an enlarged root system (unpublished data). For example, water use efficiency displayed a positive MPH in plants of TH3/12 genotypes, whereas a negative MPH was detected in TH21/2 plants. These water-use patterns differed markedly from the TH17/17 genotype, these hybrid plants evaporated water to the same extent as the Tordis parental plants, and this water loss was lower than that from the tetraploid PP-E 15 parental plants. These factors resulted in a negative MPH% for the trait of water use efficiency. This detected variability in water utilization provides the basis for further breeding, which is essential for overcoming these limitations to enable the wide use of short-rotation systems even under a changing climate. We should mention that higher water usage could be a positive factor in optimizing wastewater treatment efficiency of agroforestry (refer to review by Dimitriou et al., 2009 and study by Amiot et al., 2020).

### Expression of Positive Hybrid Vigor in the Concentrations of Selected Plant Hormones

In searching for a hormonal basis to triploid hybrid vigor, our results show that the expression of heterosis may be linked with increased concentrations of several hormones in the shoot meristems of THs arising from various crossing combinations. As a novel finding, all three THs expressed MPH for jasmonic acid accumulation (TH3/12: MPH:91.91; TH17/17: MPH:67.08; TH21/2: MPH:29.73%). In Table 3, the MPH value (102.19%) was significant for the jasmonate isoleucine level in the TH3/12 plants. Presently, we lack an explanation for the detected jasmonic acid response in shoots of triploid willow hybrids, especially if we consider results from proteomic and transcriptomic analysis of seedling leaf tissues of the maize hybrid, B73xMo17, and its inbred parents (Birdseye et al., 2021). These authors reported repression of jasmonic acid biosynthetic enzyme levels in that maize hybrid. Our data may therefore suggest a potential link to biotic stress tolerance in willow. The hormone results here highlight hybrid heterosis in the accumulation of another stress-related hormone, abscisic acid (ABA), and in addition to its physiologically inactive metabolites such as dihydrophaseic acid, neophaseic acid, and ABA–glucose ester. Intensive ABA metabolism was detected only in plants of the TH17/17 willow hybrids, these showing a 33% MPH for shoot height under *in vitro* growth conditions (Table 3B). Detection of ABA as positive factors in heterosis is an unexpected finding that might partly explain the negative MPH% in stomatal conductance and lower CO_2_ assimilation rate (refer to Table 2). The essential role of ABA in the movement of stomata is well-established (reviewed by Vishwakarma et al., 2017). In contrast, in stems of intraspecific hybrids of balsam poplar (*Populus balsamifera* L.), the ABA content was negatively correlated with heterosis in stem volume as well as stem dry biomass (Hu and Thomas, 2019). Ectopic expression of an ABA inactivation gene (*ZmABA8ox1b*) enhanced plant vigor in the early stages of maize seed germination in its hybrid B73/Mo17 relative to its parental inbred lines (Li et al., 2016).

Triploid hybrid plants (TH21/2) produced by the crossing of Inger plants and PP-E7 tetraploid willows share a unique hormone composition (Table 3C). In these plants, considerable hybrid vigor was expressed in natural auxins (indole-3-acetic acid: MPH207.49%; phenylacetic acid: MPH-223.51%) that was not detectable in plants of the other two willow hybrids. The indole-3-acetic acid content of stem of intraspecific hybrids of balsam poplar (*P. balsamifera*) showed a strong positive correlation with heterosis in stem volume and stem dry biomass (Hu and Thomas, 2019). Higher auxin concentrations were detected in the seedling of canola (*Brassica napus* L.) hybrids (Zhu et al., 2020). Considering the potential roles of benzoic acid and salicylic acid in heterosis, we obtained contradictory results, in that we detected MPH for concentrations of these compounds (salicylic acid: 27.72%; benzoic acid:85.75%) as part of the heterosis response in willow. By contrast, in *Arabidopsis* F1 hybrids, an increasing salicylic acid concentration diminished their heterosis (Groszmann et al., 2015). Considering the multiple roles of cytokinins in plant developmental processes, including regulation of cell division and differentiation (by Kieber and Schaller, 2018), hybrid vigor in the concentrations of various cytokinin metabolites (Table 3) could have been responsible for the physiological responses that we detected in the willow THs. Apart from cytokinin glucosides, high levels of cytokinin ribosides were recorded in the three triploid hybrid combinations (TH3/12: 24.4, TH17/17: 21.02, and TH21/2: 8.67% + 14.26% + 26.92%). In the fast-growing young shoots of TH17/17 plants, a generally very high level of precursors for cytokinin phosphates was detected (MPH 186.09%), including that of the most physiologically active cytokinin *trans*-zeatin, the *trans*-zeatin riboside monophosphate: 500%. This result is consistent with the differential accumulation of *trans*-zeatin riboside in two maize hybrids that expressed heterosis in the development of the ear inflorescence (Shi et al., 2019).

### Positive or Negative Mid-Parent Heterosis in Methane Yield During Fermentation

Short-rotation coppice willow is a lignocellulose-rich energy crop whose green and woody biomass components may be considered as substrates for the anaerobic digestion (AD) process of biomethane production (Kakuk et al., 2021; Nyári et al., 2021). Our study demonstrates that breeding triploid hybrid willow genotypes can open a way forward for applying hybrid vigor to improve the bioenergy production system. As shown in Figure 4, positive or negative MPH in the methane yield (ml methane/1 g FW organic material) was obtainable when using the woody biomass from different THs. In the fermentation of woody tissues from the TH3/12 hybrids, the positive hybrid vigor (MPH:23.25%) in methane production was correlated with the lowest acid detergent lignin (ADL) content (13.89%). The greater efficiency in methane production was evident throughout the fermentation process (Figure 3). The highest ADL ratio (19.17%) was detected in wood samples from the Inger plants. Yet, this ADL level was reduced to 14.64% in the case of TH21/2 hybrids featuring negative heterosis in methane yield (MPH: –14.33%). Concerning their chemical composition, wood tissues from the TH17/17 hybrids contain more lignin than do tissues of its parents (MPH: 15.59%); hence, fermentation of this type of woods resulted in a reduced methane yield (MPH:- 27.80%). These trends are consistent with the previous findings that methane production decreases as the lignin content increases (Herrmann et al., 2016). In this respect, a negative correlation (*r* = −0.82) was obtained when using green or woody willow biomass for methane generation (Kakuk et al., 2021). Our experimental findings focus our attention upon fermentation efficiency as a special and important breeding target. Triploid heterosis offers a novel genetic basis for the selective improvement of valuable traits.

## Concluding Remarks and Future Directions

As a unique genetic approach in tree breeding, crossing between leading willow varieties and artificial autotetraploid genotypes can generate significant hybrid vigor not only in F1 progenies but also during subsequent vegetative propagation of these hybrid plants. The production of triploid energy willow genotypes resulted in heterosis of biomass-related traits and the triploid nature, also affecting the hybrid vigor for methane yield during anaerobic fermentation. The varying degree of heterosis responses clearly depended on the combination of parental genotypes. Significant hybrid vigor, expressed as MPH% values, was recorded for the early growth rate of shoots, and CO_2_ assimilation rate. Beyond hybrid vigor terms of physiological parameters, positive MPH% could be detected in the hormonal profiles of THs. Unexpectedly, the content of jasmonic acid in shoot meristems of all the three THs evinced hybrid vigor. The various triploid hybrid genotypes differed in positive or negative MPH% with respect to stomatal conductance and water use efficiency values. We suggest that using triploid willow hybrids with negative MPH in acid detergent lignin contents could contribute to an augmented methane yield. The results of this study support the wider use of triploid heterosis for increasing the biomass productivity of energy trees grown in short-rotation plantations. Our study also emphasizes the need to initiate breeding activities that focused on enhancing those traits, which mostly determine the efficiency of biogas production technologies.

## Data Availability Statement

The original contributions presented in the study are included in the article/supplementary material, further inquiries can be directed to the corresponding author/s.

## Author Contributions

DD designed the experiments and performed the crossings, and field and *in vitro* experiments, conducted the data analysis, and prepared the manuscript. AC did the phenotyping, field experiments, data analysis. KT carried out the embryo culture and *in vitro* experiments. LS conducted the data analysis. ZZ collected data and performed the statistical analysis. GF analyzed the data and prepared the manuscript. PP performed the photosynthetic measurements. PB contribute to statistical analysis. ZC performed the chemical analysis. RV and PD performed the hormone analysis. JS performed the wood composition analysis. ZB and KK performed the biogas fermentation experiments. All authors contributed to the article and approved the submitted version.

## Conflict of Interest

The authors declare that the research was conducted in the absence of any commercial or financial relationships that could be construed as a potential conflict of interest.

## Publisher’s Note

All claims expressed in this article are solely those of the authors and do not necessarily represent those of their affiliated organizations, or those of the publisher, the editors and the reviewers. Any product that may be evaluated in this article, or claim that may be made by its manufacturer, is not guaranteed or endorsed by the publisher.
